# Analysis of peptide-SLA binding by establishing immortalized porcine alveolar macrophage cells with different SLA class II haplotypes

**DOI:** 10.1186/s13567-018-0590-2

**Published:** 2018-09-21

**Authors:** Quy Van Chanh Le, Thong Minh Le, Hye-Sun Cho, Won-Il Kim, Kwonho Hong, Hyuk Song, Jin-Hoi Kim, Chankyu Park

**Affiliations:** 1 Department of Stem Cell and Regenerative Biotechnology, Konkuk University, Gwangjin-gu, Seoul, South Korea; 2 College of Veterinary Medicine, Chonbuk National University, Iksan, Republic of Korea

## Abstract

Primary porcine alveolar macrophages (PAM) are useful for studying viral infections and immune response in pigs; however, long-term use of these cells is limited by the cells’ short lifespan. We immortalized primary PAMs by transfecting them with both *hTERT* and *SV40LT* and established two immortalized cell lines (iPAMs) actively proliferating even after 35 passages. These cells possessed the characteristics of primary PAMs, including strong expression of swine leukocyte antigen (SLA) class II genes and the inability to grow anchorage-independently. We characterized their SLA genes and subsequently performed peptide-SLA binding assays using a peptide from porcine circovirus type 2 open reading frame 2 to experimentally measure the binding affinity of the peptide to SLA class II. The number of peptides bound to cells measured by fluorescence was very low for PK15 cells (7.0% ± 1.5), which are not antigen-presenting cells, unlike iPAM61 (33.7% ± 3.4; *SLA*-DQA*0201/0303, DQB1*0201/0901, DRB1*0201/1301) and iPAM303 (73.3% ± 5.4; SLA DQA*0106/0201, DQB1*0202/0701, DRB1*0402/0602). The difference in peptide binding between the two iPAMs was likely due to the allelic differences between the SLA class II molecules that were expressed. The development of an immortal PAM cell panel harboring diverse SLA haplotypes and the use of an established method in this study can become a valuable tool for evaluating the interaction between antigenic peptides and SLA molecules and is important for many applications in veterinary medicine including vaccine development.

**Electronic supplementary material:**

The online version of this article (10.1186/s13567-018-0590-2) contains supplementary material, which is available to authorized users.

## Introduction

Peptide presentation to T helper (CD4^+^) and cytotoxic T lymphocytes (CTLs, CD8^+^) by major histocompatibility complexes (MHCs) is essential for adaptive immunity in vertebrates [1–3]. MHC class I and II molecules represent intracellular and extracellular peptides, respectively [4]. Studying the formation of peptide-MHC complexes is important to extend our knowledge on epitope selection for vaccine development, the evolutionary mechanisms of pathogen for immune evasion, and the influence of MHC polymorphisms on the disease resistance and susceptibility [5–9].

It has been suggested that the absolute binding capacity of peptides to MHCs is a good indicator for immunogenicity [10]. Therefore, various bioinformatic tools and resources that can predict the binding affinity of peptides to MHC molecules have been reported, including MHC-PPM, NetMHCpan-3.0, NetMHCII, and NetMHCIIpan [11–13]. However, the predicted interactions and their effects on the immune system need to be experimentally validated [14, 15]. For this purpose, several methods have been developed, including cell-free systems using purified MHC molecules [16, 17], artificial antigen processing systems by transformation of *E. coli* or yeast with recombinant MHC class II α and β chains [18–20], and mammalian cell systems using native MHC molecules on the cell surface [21, 22].

Studies to address the interaction between MHCs and epitopes have been limited for nonhuman species such as pigs. The development of disease resistance and effective vaccines against major infectious diseases in pigs is a critical issue in large-scale pig farming as diseases cause significant losses in productivity [23, 24]. However, interactions between swine leukocyte antigens (SLAs) and the epitopes of viral pathogens have not been extensively studied.

To improve their capacity for antigen presentation, MHC genes have evolved to expand their diversity [9]. For example, 2165 *HLA*-*DRB1*, 1196 *HLA*-*DQB1*, and 94 *HLA*-*DQA1* alleles are currently reported for human leukocyte antigens (HLAs) [25]. On the other hand, 99 *SLA*-*DRB1*, 53 *SLA*-*DQB1*, and 26 *SLA*-*DQA* alleles are currently reported for SLAs [26], suggesting that further studies are needed to improve our understanding of the immune system of domestic animals.

Monocytes and macrophages play critical roles in the immune response through phagocytosis, antigen presentation, and cytokine secretion [27, 28]. Simultaneously, macrophages serve as target cells for the replication of major pathogenic viruses in pigs, such as porcine respiratory and reproductive syndrome virus (PRRSV), African swine fever virus (ASFV), classical swine fever virus (CSFV), porcine circovirus 1 (PVC-1), and porcine circovirus 2 (PCV-2) [29–32].

A pig alveolar macrophage (PAM) cell line showed the ability to support the replication of porcine adenovirus (PAV), vaccine virus (VV), bovine adenovirus (BAV), parainfluenza virus, herpes simplex virus (HSV), swine poxvirus, African swine fever virus (ASFV), classical swine fever virus (CSFV), pseudorabies virus (PRV), and vesicular stomatitis virus (VSV) [33]; therefore, it can be a good model for studying virus-host interactions, including the formation of peptide-SLA complexes. However, because of difficulties involving cell preparation, limited lifespan, and experimental variation from different source animals, stable cell lines with the phenotypic characteristics of primary PAMs are being considered as potential alternatives.

The use of simian vacuolating virus 40 large T (SV40LT) antigen and human telomerase (hTERT) has been proven to be a simple and reliable method to immortalize primary cells [34]. In this study, we established two stable PAM cell lines with known MHC haplotype information and used them to measure the binding affinity between MHC class II molecules and a peptide from PCV2. Our strategy can be used to evaluate the binding affinity of various pathogenic peptides to SLA class II molecules, which can be useful for immunogenetic applications in pigs.

## Materials and methods

### Collection of porcine alveolar macrophages and cell culture

PAMs were isolated from ten 10-week-old clinically healthy Yorkshire pigs raised at a local pig farm. Animals were humanely euthanized and the lungs were collected for cell isolation. Phosphate buffer was used for bronchoalveolar lavage through a conventional method [35]; the bronchoalveolar lavage fluid was subsequently filtered. PAMs were collected by centrifugation at 400 ×* g* for 5 min and were cultured in six-well plates in Roswell Park Memorial Institute (RPMI) 1640 medium (Hyclone, UT, USA) supplemented with 10% fetal bovine serum (FBS) and a 1% penicillin–streptomycin-gentamycin antibiotic mixture (Gibco, NY, USA) at 37 °C and 5% CO_2_ in an incubator. The adherent alveolar macrophages were cultured for 48 h and frozen in cell freezing media (RPMI 1640; 20% FBS; 10% dimethyl sulfoxide, DMSO) overnight in an isopropanol box at −80 °C and subsequently stored in liquid nitrogen until use. The PAM 3D4/21 cell line was purchased from the American Type Culture Collection (ATCC CRL-2843) and was cultured in RPMI 1640 supplemented with 10% FBS and the antibiotic mixture. The fibroblast PK15 cell line (ATCC CCL-33) was cultured in DMEM with the same concentration of FBS and antibiotic mixture. All animal procedures were approved by the Institute of Animal Care and Use Committee of Konkuk University.

### SLA typing

Genomic DNA was prepared from cells using an RNA/DNA mini kit (Qiagen, Hilden, Germany) according to the manufacturer’s protocol. Typing of five SLA genes, *SLA1*, *SLA2*, *SLA*-*DQB1*, *SLA*-*DRB1*, and *SLA*-*DQA* was performed using genomic sequence-base typing (GSBT) as previously described [36–40]. Sequencing of PCR products for each locus were performed using ABI PRISM BigDyeTM Terminator Cycle Sequencing Kit (Applied Biosystem, USA) following the manufacturer’s protocol. The sequencing results were analyzed by the CLC Workbench (CLC Bio, Arhus N, Denmark).

### Immortalization of PAMs

Primary alveolar macrophages were transfected with pBABE-*SV40LT*-Puro [41] and pBABE-*hTERT*-Puro [42] using PolyMag kit (Chemicell, Berlin, Germany) following the manufacturer’s protocol. After 48 h, cells were subjected to selection using 2 µg/mL puromycin (Sigma-Aldrich, lnc, Germany) for 4 weeks. Cells were continuously cultured until passage 35. The cells were subsequently frozen in cell freezing media without antibiotics in cryogenic vials (Nalgene, NY, USA) at −80 °C for 24 h before storing in liquid nitrogen.

### Confirming hTERT and SV40LT integration by PCR

Genomic DNA was isolated from 5 × 10^5^ cells using an RNA/DNA mini kit (Qiagen, Hilden, Germany) according to the manufacturer’s protocol. PCR was performed in a 20 µL reaction containing 100 ng of genomic DNA, 0.5 µM primer pairs of *hTERT* or *SV40LT*, 0.5 U of Super-Therm DNA polymerase (JMR Holdings, Kent, UK) in 1.2× PCR buffer (1.5 mM MgCl_2_), and 0.1 mM dNTPs using the T3000 Thermocycler (Biometra, Göttingen, Germany). The primer pairs hTERT-F (5′-GCCGAGACCAAGCACTTCCTCTACT-3′) and hTERT-R (5′-GCAACTTGCTCCAGACACTCTTCCG-3′), and SV40LT-For (5′-GATGGCTGGAGTTGCTTGGCTACAC-3′) and SV40LT-Rev (5′ GCCTGAAATGAGCCTTGGGACTGTG-3′) produce 778-bp and 858-bp amplicons, respectively. The PCR profile consisted of an initial denaturation cycle of 5 min at 95 °C, 35 cycles of 30 s at 94 °C, 30 s at 63 °C, 45 s at 72 °C, and a final extension of 5 min at 72 °C. The PCR products were analyzed in a 1% agarose gel through electrophoresis.

### Determination of cell growth curve and soft agar growth

The immortalized PAMs (2 × 10^5^ cells/well) were seeded in a six-well plate containing RPMI 1640 (Hyclone) with 15% FBS and 5 µg/mL gentamicin. The cells were trypsinized at 24, 48, 72, 96 h post-seeding, and then counted using the trypan blue exclusion method [43]. The number of cells at each time point was plotted. To measure cell anchorage-independent proliferation, which is a typical characteristic of immortalized cells, 1 × 10^4^ cells/well of iPAM 303 and iPAM61 were cultured in a six-well soft agar dish prepared with 0.8% agar and 0.7% agarose in the bottom and upper layers, respectively, for 2 weeks. Cells were fed with cell culture media every 2 days. Colony formation was checked under a light microscope (Model BX51TF, Olympus, Tokyo, Japan).

### Immunohistochemistry

Cells were seeded on glass cover slips (Waner Instruments, LLC, Hamden, USA) in a six-well plate at a density of 1.2 × 10^5^ cells/well and cultured for 24 h. Subsequently, the cover slips were washed in 1 × phosphate-buffered saline (PBS) and fixed with 4% paraformaldehyde for 15 min at room temperature. Cells were washed with 1× PBS and then incubated in 1× PBS with 5% bovine serum albumin (BSA; Sigma-Aldrich, St. Louis, MO, USA) for 30 min. Subsequently, the cells were incubated with mouse anti-SLA-DRB1 monoclonal antibody (1:250 dilution, Bio-Rad, California, USA) in 1× PBS containing 2.5% BSA for 2 h. For visualization, cells were incubated with a goat anti-mouse antibody conjugated with Alexa Fluor 568 (1/500 dilution; Invitrogen, Massachusetts, USA) in 1× PBS containing 2.5% BSA for 1 h. For covering with a glass cover slip, VECTASHIELD Mounting Medium with DAPI (Vector, Burlingame, CA, USA) was used. Samples were analyzed under a fluorescence microscope (Model BX51TF, Olympus, Tokyo, Japan).

### Analysis of peptide binding to SLA class II molecules

For the peptide binding assay using the immortalized PAMs, we chose a 15-amino-acid peptide (RSHLGQILRRRPWLV, indicates the core binding region) derived from the open reading frame (ORF) 2 of porcine circovirus type 2 (PCV2), which encodes a major capsid protein and is known to activate the SLA class II-restricted immune response [44, 45]. The selected sequence was synthesized via solid phase peptide synthesis, conjugated with biotin at the N-terminal, and purified by high performance liquid chromatography (HPLC) using a commercial service (GeneScript, Piscataway Township, NJ, USA). For analysis through flow cytometry, cells were harvested using trypsin–EDTA and pellets were resuspended in 100 uL of RPMI 1640 medium (Hyclone) containing 5% FBS and pH-adjusted to 7.2. Cells (1 × 10^5^) were incubated with the biotinylated peptide (50 µM) in microcentrifuge tubes for 5 h at 37 °C and 5% CO_2_. The cells were centrifuged at 400 × *g* for 5 min and then washed twice with the fluorescence-activated cell sorting (FACS) buffer (1× PBS, 1% FBS, 0.05% sodium azide). Cell pellets were resuspended in a 1:40 dilution of streptavidin-FITC conjugate (Thermo Fisher Scientific, Massachusetts, USA) in the wash buffer and then incubated on ice for 30 min. Subsequently, the cells were washed twice with the wash buffer and then finally resuspended in 500 μL FACS buffer. Fluorescence signals from the peptide-SLA class II complexes was quantified by FACScalibur (BD Bioscience, San Jose, CA, USA) with CellQuest software. The results were calculated and analyzed by one-way analysis of variance (ANOVA) and Student’s *t*-test.

## Results

### Generation of immortalized porcine alveolar macrophages (PAMs)

**Figure 1:**
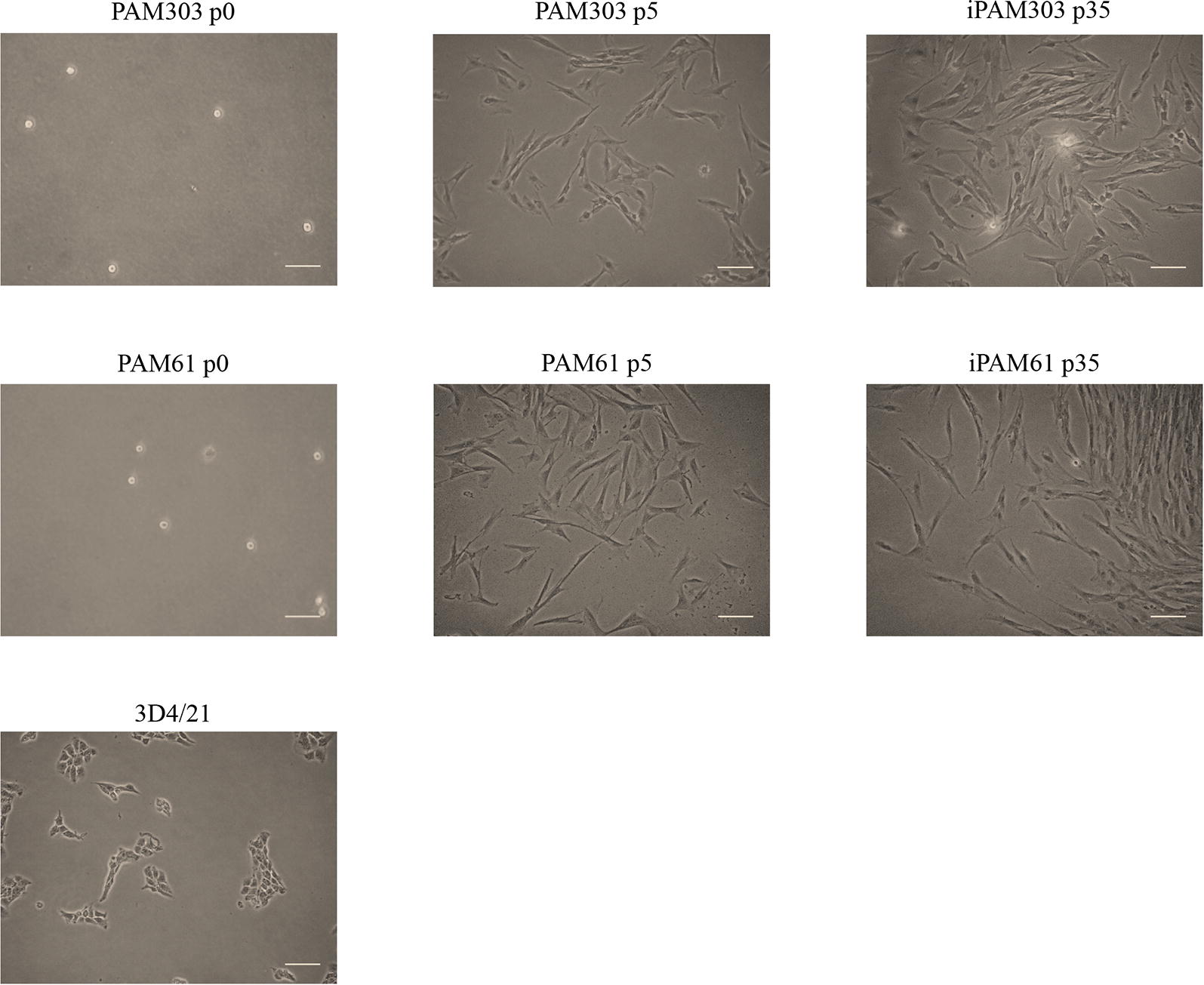
**Morphological comparison of immortalized porcine alveolar macrophages (iPAMs) at different passages.** The names of cells with the corresponding passage numbers are indicated on top. Scale bar: 100 µm.

**Figure 2:**
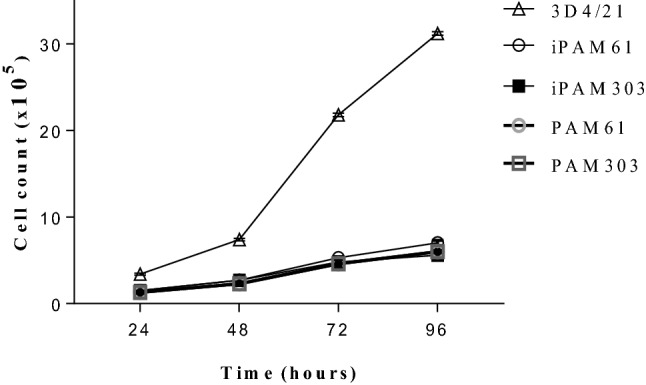
**Growth curves of immortalized porcine alveolar macrophages (iPAMs), early stage primary PAMs and 3D4/21.** The primary PAM cells at passage 4 and iPAMs at passage 35 are used. Duration of culture is indicated in the x-axis.

We evaluated the phenotypes of the PAMs transfected with *hTERT* and *SV40LT* at passage 35. The PAMs that were round-shaped at passage 0 became spindle-shaped when they attached to the culture plates as early as passage 5 (Figure 1). The morphology of immortalized PAMs (iPAMs) were very similar to that of early passage primary PAMs. The results were consistent for both iPAM61 and 303. However, ATCC 3D4/21 was differently shaped and smaller than both iPAM61 and 303. The cells attached to the surface of the culture tube at 24 h. The number of healthy cells were counted every 24 h. The cells proliferated exponentially without showing signs of senescence unlike the primary PAMs, which stopped dividing around passage 15. Growth curves of the iPAMs at passage 35 together with primary PAMs and 3D4/21 are shown in Figure 1. The patterns are similar among all compared cells except 3D4/21 which showed much faster proliferation, supporting consistent characteristics between early stage primary PAMs and iPAMs (Figure 2).

**Figure 3:**
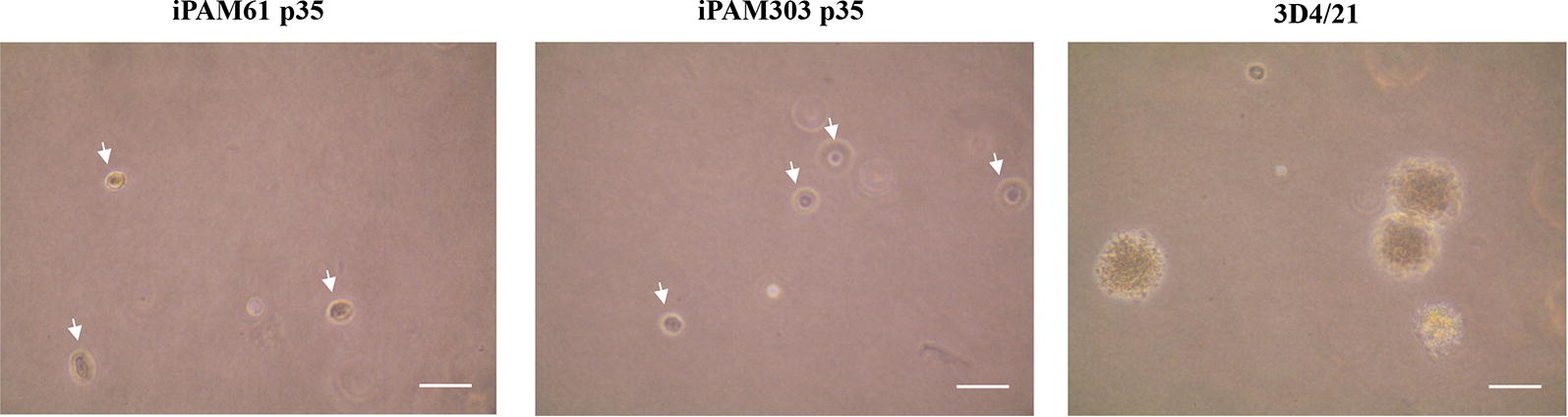
**Anchorage-independent growth of immortalized porcine alveolar macrophages (iPAMs) in soft agar.** The names of cells are indicated on top iPAM61 and 303 are from passage 35 (p35). Arrowheads indicate single cells. Scale bar: 50 µm.

Anchorage-independent growth is the ability of transformed cells to grow without requiring a solid surface, and this was tested for iPAM61, iPAM303, and 3D4/21 cells using a soft agar clonogenic assay. While 3D4/21 cells formed numerous colonies, iPAM303, iPAM61, and the primary PAM cells did not form colonies even after 2 weeks on soft agar (Figure 3); this suggests that the characteristics of iPAM61 and 303 are closer to that of primary PAMs.

**Figure 4:**
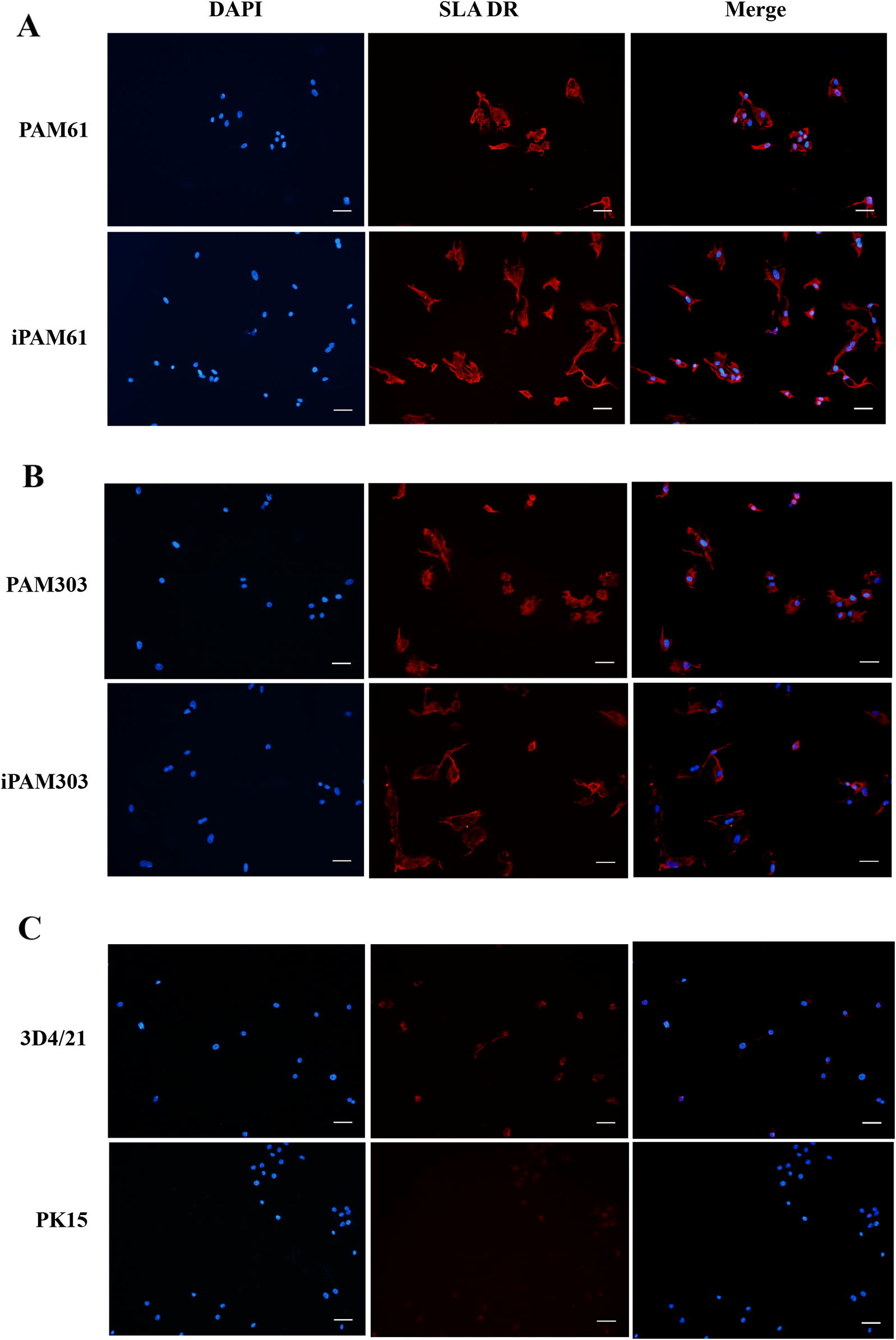
**Comparison of SLA-DR expression levels by immunohistochemical analysis.** Primary PAMs, PAM61 (**A**) and PAM303 (**B**); immortalized PAMs, iPAM61 (**A**) and iPAM303 (**B**). Control cells, 3D4/21 (**C**) and PK15 (**C**). The first, second, and third columns show the results from DAPI staining with anti-SLA-DR specific antibody. The third column indicates merged images with ×20 magnification. Scale bar: 100 µm.

The expression of MHC class II genes is an important characteristic of professional antigen-presenting cells (APCs) such as macrophages. We examined the presence of SLA-DR molecules in iPAM61, iPAM303, and 3D4/21 cells using an anti-SLA-DRB1 antibody (Figures 4A and B). Positive signals indicating the strong expression of *SLA*-*DR* were observed in iPAM303 and iPAM61, similar to that observed in primary PAMs before passage 5; on the other hand, the signal was barely detectable in 3D4/21 cells and pig fibroblasts, PK15, which is not an APC (Figure 4C). These results indicate that iPAM61 and 303 more closely resemble primary PAMs than publicly available immortalized PAM cells, including 3D4/21.

The integration and expression of *hTERT* and *SV40LT* in iPAM61 and 303 was evaluated by PCR. Both iPAM61 and 303 produced 778-bp amplicons corresponding to *hTERT* (Additional file 1). However, the amplicons for *SV40LT* was not observed in both cells. Consistently, we also failed to amplify *SV40LT* from the plasmid construct itself (Additional file 2), indicating that our inability to amplify the gene may be attributed to a structural constraint in the sequence. A similar phenomenon was also reported in a previous study [33].

### Genetic polymorphisms of SLA genes from three immortalized PAM cells

SLA GSBT was carried out for *SLA1, SLA2, SLA*-*DQA, SLA*-*DQB1*, and *SLA*-*DRB1* in 3D4/21, PAM61, and PAM303. For MHC class I genes, four alleles were each identified for *SLA1* and *SLA2*; for MHC class II genes, four alleles were each identified for *SLA*-*DQA* and *SLA*-*DQB1*, while 5 alleles were identified for *SLA*-*DRB1* (Table 1). PAM61 was homozygous for both *SLA1* and *SLA2* and heterozygous for all three MHC class II genes typed in this study. PAM303 was heterozygous for all five genes, showing high genetic variability in MHC genes. Allele redundancy among three PAM cells was low, indicating that the results of the MHC-peptide binding assays using these cells can be compared and evaluated for differences in the binding affinity of a candidate peptide to MHC molecules.

**Table 1:** **SLA typing for the three pulmonary alveolar macrophage cells used in this study**

Cells	Class I		Class II		
	SLA1	SLA2	DQA	DQB1	DRB1
3D4/21	040101	040201	0203	0201	0501
PAM61	0402	0801	0201/0303	0201/0901	0201/1301
PAM303	0801/0810	0502/1201	0106/0201	0202/0701	0402/0602

### Determination of binding affinity between a PCV2 derived peptide and SLA class II molecules with defined haplotypes in immortalized PAM cells

Understanding the binding affinity between pathogenic peptides and MHC class II molecules is critical for predicting the strength of adaptive immune responses against peptides derived from a specific pathogen. To develop a method for measuring the binding affinity of any candidate peptide antigens to SLA-DR or SLA-DQ molecules in pigs, we established the immortalized PAM cells, iPAM61 and iPAM303 with characterized MHC haplotype information, and exposed them to a synthetic biotinylated peptide (Biotin-RSHLGQILRRRPWLV) corresponding to amino acid positions 16 to 30 of PCV2 ORF2. The peptide was reported to induce specific immune responses against PCV2 with no knowledge on the binding preference for either *SLA*-*DQ* or *SLA*-*DR* [44]. Because SLA class II-deficient APCs are currently not available, we used pig fibroblast cells, PK15, as a negative control to indirectly compare the signals from the specific binding of the peptide to MHC class II molecules. The expression of *SLA*-*DR* was almost undetectable in PK15 (Figure 4C), verifying that the signals observed in the PAMs are likely to be from the peptides bound to SLA-DR molecules.

**Figure 5:**
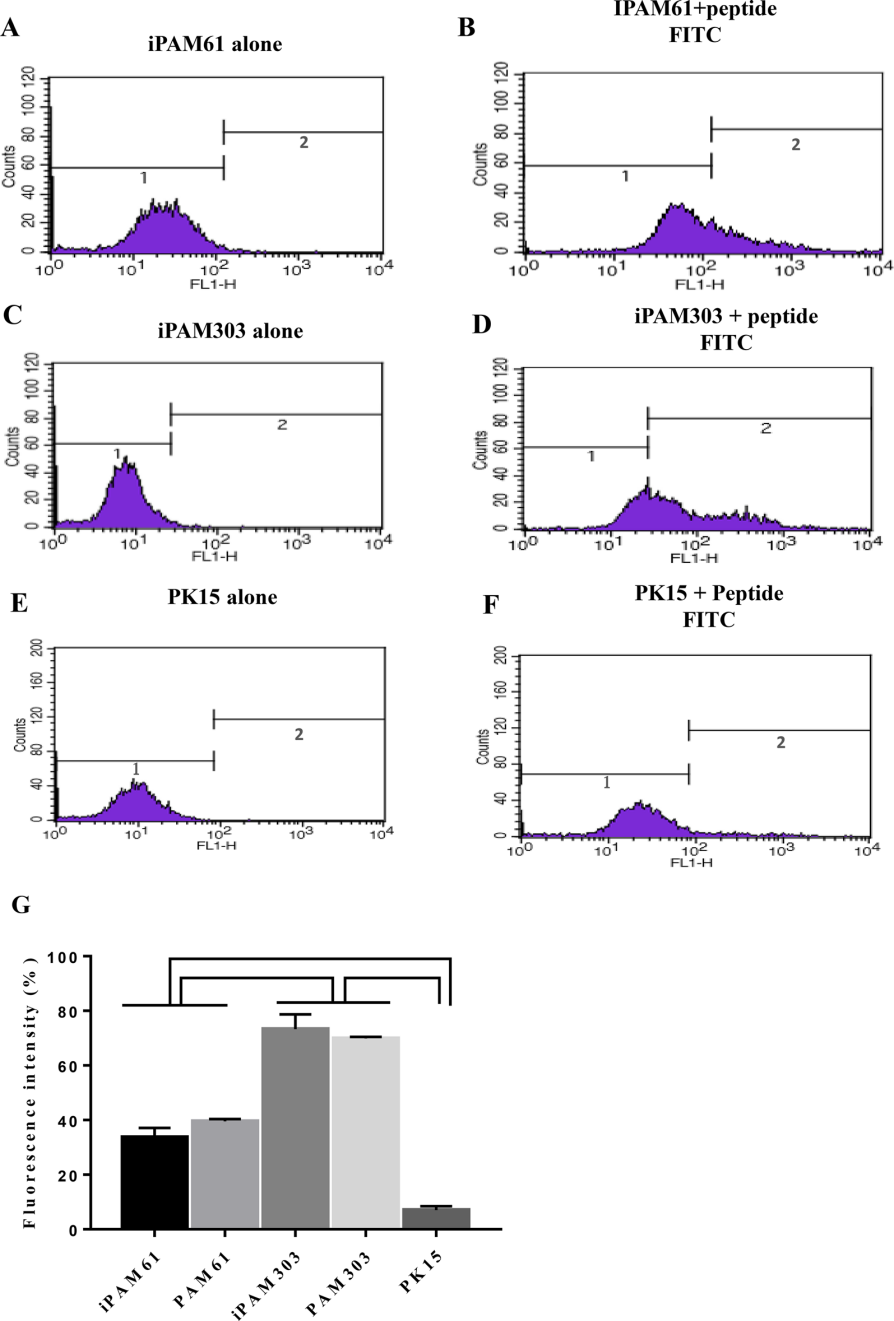
**Comparison of the efficiency of PCV2 ORF2 peptide binding to SLA class II molecules among different cells.** The fluorescence levels from SLA-peptide complexes were compared among iPAM61, iPAM303, and PK15. The left (**A**, **C**, **E**) and right (**B**, **D**, **F**) columns indicate groups without and with biotin-labeled peptides, respectively. Fluorescence levels from the SLA-peptide complexes of PAM61 and PAM303 were shown in Additional file 3. Comparison of fluorescence signals from the SLA-peptide complexes of all three types of PAMs were shown (**G**). Comparisons indicated on top with brackets differ with *P* < 0.001.

The level of fluorescence corresponding to the number of peptides bound to PAMs was measured by flow cytometry after incubating biotin-labeled peptides with iPAMs for 5 h (Figure 5). Specific signals were very low for PK15 (7.0% ± 1.5), in contrast with the observed specific signals for iPAM and PAM cells.

Furthermore, the signal intensity was significantly higher in iPAM303 (73.3% ± 5.4) than in iPAM61 (33.7% ± 3.4), indicating a difference in the number of bound peptides. The signal intensities from primary PAMs were 69.9 ± 0.6% and 39.5 ± 0.9% for PAM303 and PAM61, respectively, which are similar to those of iPAMs (Additional file 3). This result suggests that the peptide bound to SLA-DR or SLA-DQ molecules of iPAM303 (SLA DQA*0106/0201, *SLA*-DQB1*0202/0701, DRB1*0402/0602) either with higher affinity or with more efficiency than those of iPAM61 (*SLA*-DQA*0201/0303, *SLA*-DQB1* 0201/0901, DRB1*0201/1301).

## Discussion

We carried out a small-scale study to demonstrate the successful determination of the binding affinity between a peptide of PCV2 ORF2 and MHC class II molecules in the porcine immune system. We showed differences in the antigen binding affinity of the peptide to different MHC class II haplotypes, which probably reflects the efficiency of antigen presentation. Thus, for the first time, we showed results that highlighted the difference in binding affinity of a specific epitope to SLA molecules.

The association of antigenic peptides and MHC molecules is a saturable, low-affinity interaction (dissociation constant [K_d_] ~10^−6^ M) with a slow on rate and a very slow off rate [46, 47]. Once bound, peptides may stay associated for hours to days [48]. The more stable peptide-MHC complexes persist longer on the surfaces of antigen-presenting cells to ensure productive interactions with antigen-specific T cells and not with lower affinity complexes [49].

Several methods have been proposed to determine the affinity of peptides to MHC molecules, including assays with purified MHC proteins and whole-cell peptide binding assays [16, 17, 21, 22]. The whole-cell peptide binding assay presents complications in its difficulty to differentiate signals of nonspecific peptide binding to other cell-surface molecules and the degradation of peptides by cell-surface proteases in addition to the binding with the MHC itself. However, the whole-cell assay can also have advantages over other methods in estimating peptides-MHC complex for diverse MHC alleles at the same time if a panel of cells can be prepared without needing cloning and expression for a large number of MHC alleles. Additionally, peptide-treated cells can be further used to measure the efficacy of T cell activation, which is the conclusive indicator of a T cell-mediated immune response. A panel of immortalized PAMs with enough cell lines covering diverse MHC alleles can be a useful tool to screen peptides for a dominant epitope for vaccine development, as well as to improve disease resistance by adjusting the frequency of MHC alleles in animal populations.

In the whole-cell assay, the accurate assessment of peptide-MHC binding affinity needs to evaluate results not only from the peptide-MHC binding reaction but also from the peptide-MHC class II-deficient mutant cells and blocking antibody reactions to evaluate the contribution of nonspecific signals [50, 51]. However, we were unable to obtain antibodies to block the peptide-MHC complex formation, and thus the lack of currently available tools for SLA prevented us from directly measuring nonspecific signals. Therefore, we indirectly estimated the degree of nonspecific peptide binding to pig cells using a fibroblast cell line, PK15. The signal intensity from the peptide-SLA complex was similar to the expression level of *SLA*-*DR* (Figures 5E and F), indicating that the nonspecific contribution to peptide-SLA complex was negligible. Although we cannot disregard the possibility of nonspecific binding to receptors unique to PAMs rather than fibroblast cells, the difference in signals between iPAM61 and 303 supports otherwise. Therefore, our results indicate that the strategy used in this study is applicable for determining the binding affinity of a specific peptide to SLA molecules; although, establishing more cell lines with diverse MHC haplotypes is necessary.

The 15-amino-acid peptide used in this study is not suitable for presentation by MHC class I molecules because the binding capacity of MHC class I molecules are limited to peptides that are 8 to 10-amino acids long [52]; therefore, we only considered the effect of MHC class II binding. There are two major MHC class II molecules in the SLA system, *SLA*-*DQ* and *SLA*-*DR*. However, we were unable to tell at this point if the binding of the peptide was restricted to either DQ or DR, or if it binds to both molecules. Further studies and development of relevant tools are necessary to fully elucidate this.

Although we transfected cells with both *hTERT* and *SV40LT*, the inability to amplify *SV40LT* from the iPAMs indicates that overcoming replicative senescence in iPAMs may resulted from hTERT only. However, we were unable to entirely exclude the possibility of *SV40LT* as a causal role because of the amplification failure of *SV40LT* even from the plasmid construct itself (Additional file 2). Evaluating the molecular pathways associated with *SV40LT* overexpression in mammalian cells may help to illuminate that.

Different *MHC* alleles are associated with peptide binding repertoires of different sizes, affinity, and immunogenicity [53]. The aim of this study was to establish and evaluate the potential of our strategy for estimating the binding affinity of peptides to SLA molecules. The results using more immortalized PAMs, which are currently in progress, may lead us to a deeper understanding of the characteristics of the different alleles of SLA class II genes and important disease-related peptides in pigs. This approach could also be applied for other domestic animal species.

## Additional files

**Additional file 1.** **PCR amplification of hTERT from genomic DNA of immortalized PAM cells.** The results of PCR (A) and reverse transcription (RT) PCR (B) are indicated. The names of the cell lines are indicated on top. “+”, positive control plasmid; “−”, negative control. GAPDH was used as the control for RT-PCR. Product sizes are indicated by the arrows.

**Additional file 2.** **PCR amplification of SV40LT from genomic DNA of iPAM cells (iPAM303 and iPAM61).** The SV40LT-containing plasmid was used as a positive control. The expected size (858 bp) is indicated by an arrow.

**Additional file 3.** **Comparison of the efficiency of PCV2 ORF2 peptide binding to SLA class II molecules among two early primary PAM cells.** The fluorescence levels from SLA-peptide complexes were compared among PAM303 and PAM61. The left (A, C) and right (B, D) columns indicate groups without and with biotin-labeled peptides, respectively. The fluorescence levels from SLA-peptide complexes of PAM61 and PAM303 differ with *P* < 0.001 (E).

## Abbreviations

PAM: porcine alveolar macrophages
iPAMs: immortalized cell lines
SLA: swine leukocyte antigen
PCV2: porcine circovirus type 2
ORF2: open reading frame 2
MHC: major histocompatibility complexes
ATCC: American Type Culture Collection
GSBT: genomic sequence-base typing
FACS: fluorescence-activated cell sorting
