# Supplementary figures and images for: Analysis of peptide-SLA binding by establishing immortalized porcine alveolar macrophage cells with different SLA class II haplotypes
Source: Vet Res. 2018 Sep 21;49:96. doi: 10.1186/s13567-018-0590-2 (PMC6151021; doi:10.1186/s13567-018-0590-2)

## Slide 1
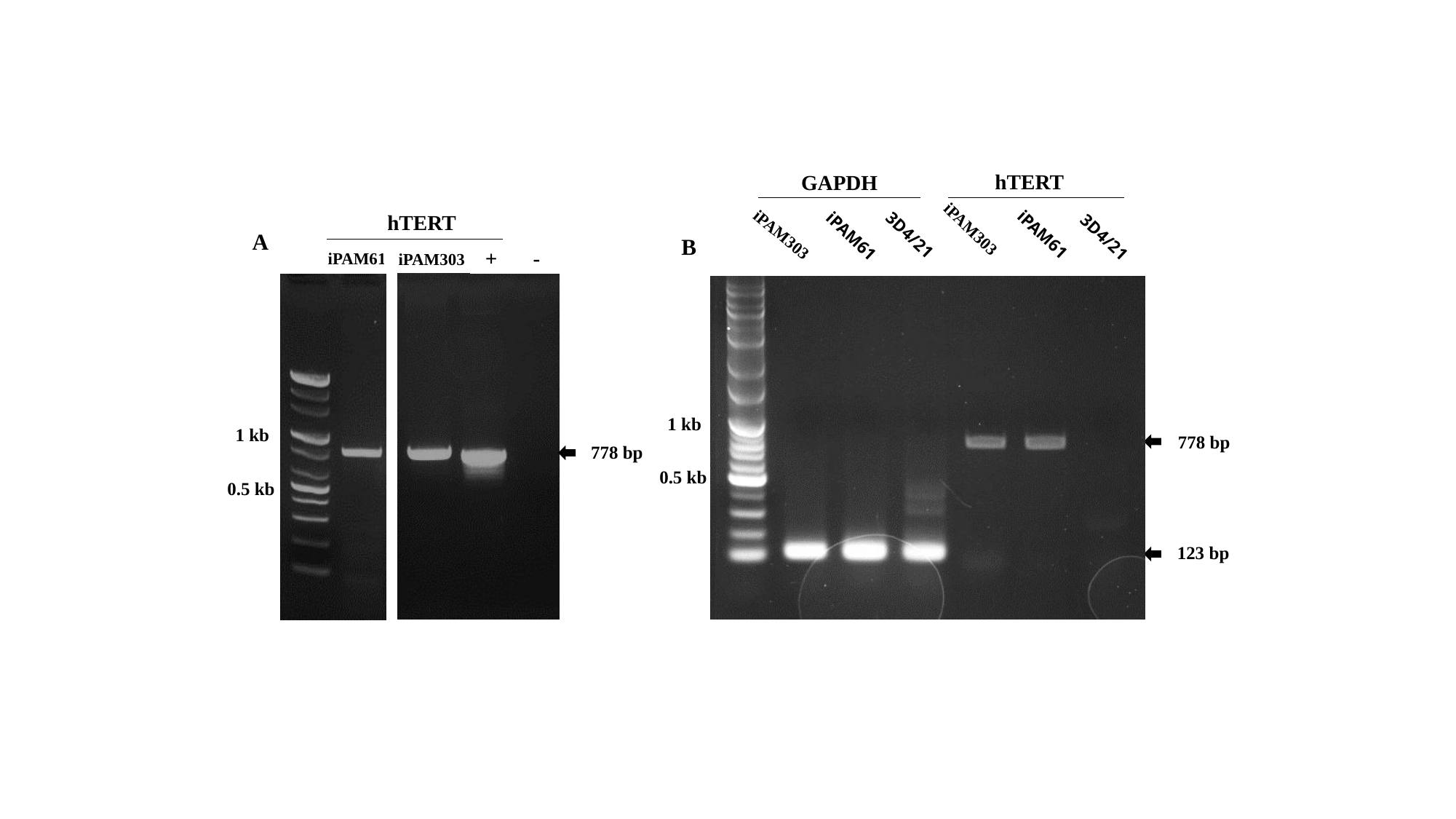

hTERT
GAPDH
iPAM303
iPAM61
3D4/21
iPAM303
iPAM61
3D4/21
-
+
iPAM303
 iPAM61
hTERT
778 bp
778 bp
123 bp
A
B
1 kb
1 kb
0.5 kb
0.5 kb

Supplement: Supplementary file 1 — Additional file 1. PCR amplification of hTERT from genomic DNA of immortalized PAM cells. The results of PCR (A) and reverse transcription (RT) PCR (B) are indicated. The names of the cell lines are indicated on top. “+”, positive control plasmid; “−”, negative control. GAPDH was used as the control for RT-PCR. Product sizes are indicated by the arrows. [file 13567_2018_590_MOESM1_ESM.pptx]

## Slide 1
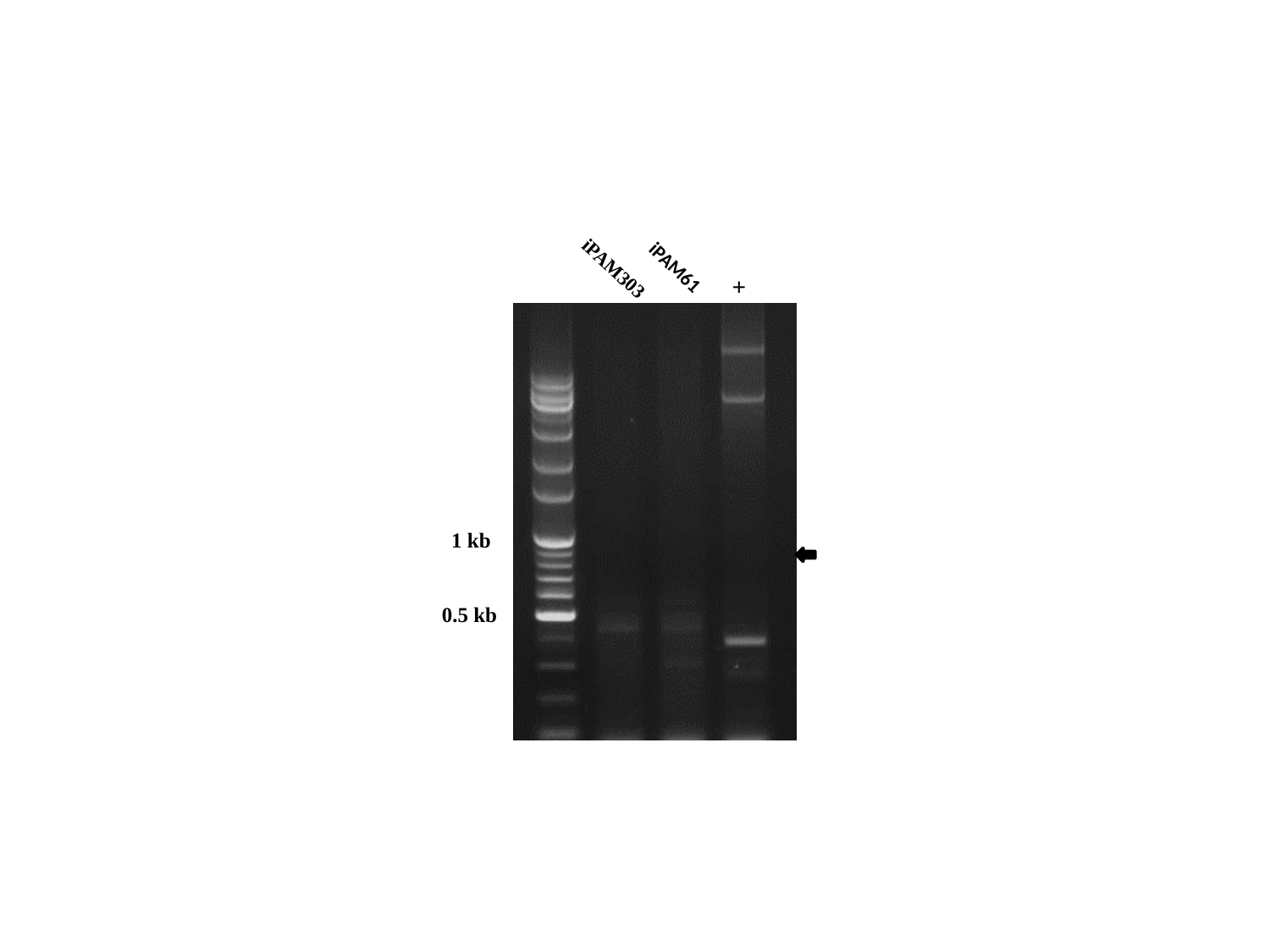

iPAM61
iPAM303
+
1 kb
0.5 kb

Supplement: Supplementary file 2 — Additional file 2. PCR amplification of SV40LT from genomic DNA of iPAM cells (iPAM303 and iPAM61). The SV40LT-containing plasmid was used as a positive control. The expected size (858 bp) is indicated by an arrow. [file 13567_2018_590_MOESM2_ESM.pptx]

## Slide 1
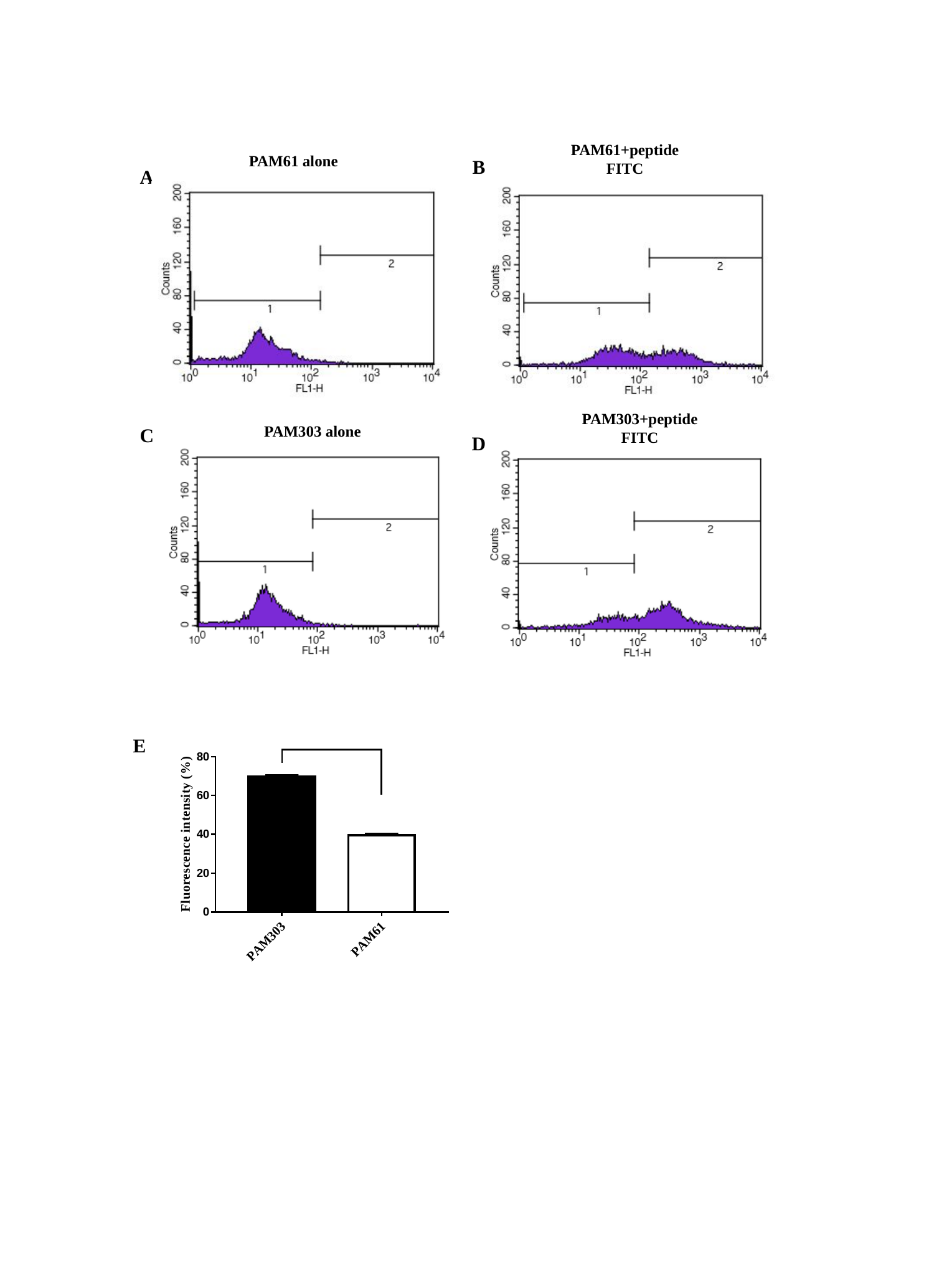

PAM61+peptide FITC
PAM61 alone
B
A
PAM303+peptide FITC
PAM303 alone
C
D
E

Supplement: Supplementary file 3 — Additional file 3. Comparison of the efficiency of PCV2 ORF2 peptide binding to SLA class II molecules among two early primary PAM cells. The fluorescence levels from SLA-peptide complexes were compared among PAM303 and PAM61. The left (A, C) and right (B, D) columns indicate groups without and with biotin-labeled peptides, respectively. The fluorescence levels from SLA-peptide complexes of PAM61 and PAM303 differ with P < 0.001 (E). [file 13567_2018_590_MOESM3_ESM.pptx]
